# Splenic embolization for a giant splenic hemangioma in a child: a case report

**DOI:** 10.1186/s12887-018-1331-4

**Published:** 2018-11-12

**Authors:** Woosun Choi, Young Bae Choi

**Affiliations:** 1 Department of Radiology, Chung-Ang University Hospital, Seoul, Republic of Korea; 2 Department of Pediatrics, Chungbuk National University Hospital, Cheongju, Republic of Korea

**Keywords:** Splenic hemangioma, Embolization, Child

## Abstract

**Background:**

Splenic hemangioma is the most common benign tumor of the spleen. However, it remains a rare medical condition in children. Although the natural course of splenic hemangioma is slow growth, treatment for large splenic hemangiomas has been recommended due to the risk of spontaneous rupture causing life-threating hemorrhage. However, the optimal treatment for splenic hemangioma in children is unclear.

**Case presentation:**

An 11-year-old girl had an enhancing mass, 61 × 54 × 65 mm in size and numerous daughter nodules throughout the entire spleen on a contrast-enhanced computed tomography scan of the abdomen and angiography. The patient was treated by complete embolization at the distal level of splenic artery, which resulted in total splenic infarction. Treatment-related complications were thrombocytosis and postembolization syndrome, including abdominal pain and, intermittent fever below 39 °C. There were no other serious complications, including bleeding.

**Conclusion:**

Splenic embolization may be a safe and less invasive intervention for children with a large splenic hemangioma. Further studies are needed to confirm the effectiveness of our approach.

## Background

While splenic hemangioma is the most common benign tumor of the spleen, it is nevertheless a rare medical condition. Less than 100 cases of splenic hemangioma have been reported, with fewer 20 being pediatric cases [1–3]. Splenic hemangiomas are most commonly found incidentally since patients are seldom symptomatic. Although the natural course of splenic hemangioma is slow growth, treatment for large splenic hemangiomas exceeding 4 cm in size has been recommended due to the risk of spontaneous rupture causing life-threating hemorrhage [4]. However, the optimal treatment approach to splenic hemangioma including surgery or other interventions in children has been unclear. We present a pediatric case in which a giant splenic hemangioma treated with splenic embolization.

## Case presentation

**Fig. 1:**
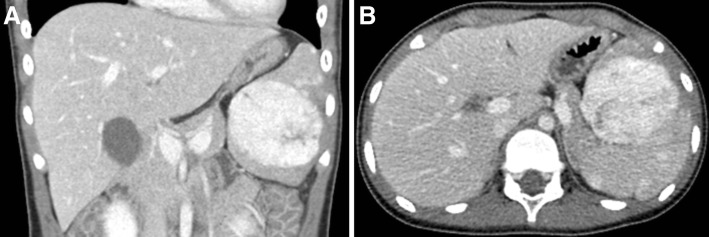
**a** and **b** Computed tomography scans shows a giant enhancing mass 61 × 54 × 65 mm in size and several subcentimeter enhancing nodules in the spleen, suggesting possible hemangiomas

An 11-year-old girl was admitted to our hospital due to abdominal pain and diarrhea of 1 week’s duration. She had no medical history of abdominal trauma or surgery. In addition, she had no travel history and there were no pets at her home. Initial assessment of vital signs showed a blood pressure of 116/70 mmHg, a heart rate of 86 beats per minute, body temperature of 36 °C, respiratory rate of 20 breaths/min, and oxygen saturation of 99%, all of which were within normal range for her age. On physical examination, she had tenderness in the right lower quadrant of the abdomen without rebound tenderness. The spleen and liver were not palpable. Laboratory examinations yielded normal results with a leukocyte count of 6,050 cells/μL, hemoglobin concentration of 13.7 g/dL, platelet count of 318,000 platelets/μL, prothrombin time of 12.3 s, and activated partial thromboplastin time of 30.6 s. Initially, she was diagnosed with acute gastroenteritis, and a contrast-enhanced computed tomography (CT) scan of the abdomen was performed to rule out acute appendicitis. A CT scan of the abdomen revealed an enhancing mass, 61 × 54 × 65 mm in size and several subcentimeter enhancing nodules in the spleen, suggesting possible hemangioma, as well as diffuse edematous wall thickening in the colon (Fig. 1). She was diagnosed with acute colitis and a giant splenic hemangioma that was found incidentally, and treated with intravenous hydration and medication for acute colitis. After the symptoms of acute colitis resolved, she received vaccinations for encapsulated bacteria including *Haemophilus influenzae* type b, *Streptococcus pneumoniae*, and *Neisseria meningitidis*.

Two weeks after completion of the vaccinations, the patient underwent splenic embolization at the interventional center by a clinically experienced interventional radiologist. The procedure was performed with the patient under general anesthesia and the electrocardiogram, blood pressure, and oxygen saturation with pulse oximetry were continuously monitored during the procedure.

**Fig. 2:**
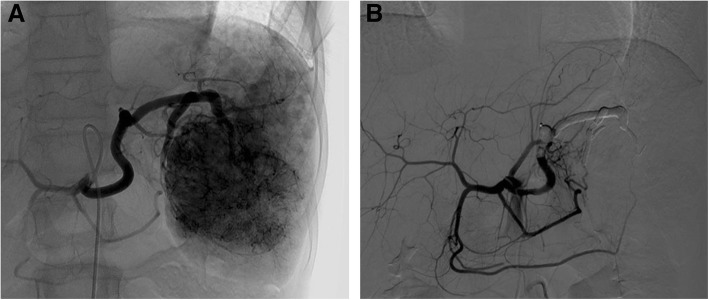
**a** Angiography of the splenic artery shows a giant hemangioma and multiple daughter nodules in the spleen. Numerous more daughter nodules were observed throughout the entire spleen on angiography. **b** Post embolization celiac angiography shows complete occlusion of the splenic artery at the distal level

The right common femoral artery was accessed under sonographic guide via the Seldinger technique and a 5-F arterial sheath was placed. A 5-Fr angiographic catheter (Yashiro Glidecath; Terumo, Tokyo, Japan) was used for access and to perform angiography of the celiac trunk and splenic artery. On celiac angiography, a giant hemangioma and multiple daughter nodules were identified in the spleen. Before performing the angiography, we had initially planned selectively embolize of the main mass and the large daughter nodules (Fig. 2a). However, numerous daughter nodules throughout the entire spleen were observed on angiography, and complete splenic artery embolization was performed. For splenic artery embolization, a 1.9-Fr microcatheter (Tellus; Asahi Intecc; Aichi, Japan) was inserted through the angiographic catheter and advanced through the distal splenic artery at the level of the hilum. Polyvinyl alcohol (Contour SE; Boston Scientific, Fremont, CA, USA) particles were initially used for splenic artery embolization and N-butyl cyanoacrylate (Histoacryl; Braun, Sempach, Switzerland) was additionally used for more complete embolization. We occluded the splenic artery at the distal level. Following embolization, angiography demonstrated complete occlusion of the splenic artery (Fig. 2b). There were no acute complications after splenic embolization including bleeding.

**Fig. 3:**
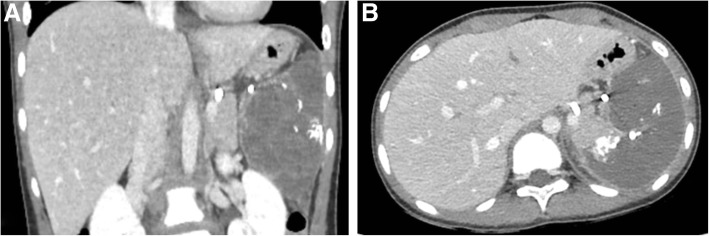
**a** and **b** Computed tomography on day 6 after embolization shows total infarction of the spleen. Embolic material at the distal splenic artery is also seen on the scans

At 4 h post-embolization, she developed mild abdominal pain, which was managed with alternating acetaminophen and ketorolac. At 12 h post-embolization, she developed intermittent fever below 39 °C, which was managed with acetaminophen. Blood and urine cultures were subsequently performed. On day 5 post-splenic embolization, hematologic studies showed thrombocytosis, with a platelet count of 502,000/μL. On day 6, a contrast-enhanced CT scan of the abdomen revealed total infarction of the spleen. There were no complications observed, including splenic abscess or bleeding (Fig. 3). On day 7, culture studies showed an absence of bacteria. The abdominal pain and fever had subsided, and the patient was discharged. During outpatient follow-up, the platelet counts peaked at 950,000/μL on day 20 post-splenic embolization and returned to normal 2 months after splenic embolization. No other complications related to the embolization, including pulmonary complications, severe infection, or portal vein thrombosis, occurred during 6 months of follow-up. The patient was prescribed daily prophylaxis with oral amoxicillin for 1 year post-embolization due to her functional asplenia.

## Discussion and conclusion

Splenic hemangioma is a rare disease, with an incidence between 0.03 and 14% in autopsy series, most commonly detected in middle-aged adults [4–6]. As hemangiomas are slow-growing benign tumors consisting of numerous blood vessels, small (< 4 cm) asymptomatic splenic hemangiomas have been managed with observation [6]. However, a previous study reported that spontaneous rupture occurs in 25% of patients and large splenic hemangiomas (≥4 cm) had a higher risk of spontaneous rupture [6]. Therefore, splenectomy has been recommended for large splenic hemangiomas due to the risk of life-threatening bleeding.

Splenic embolization was first introduced for hypersplenism treatment by Maddison in 1973 [7]. Compared with splenectomy, splenic embolization had several advantages such as less invasiveness, shorter hospital stays, decreased blood loss, and fewer operative and postoperative complications [7]. Therefore, the indications for splenic embolization included abdominal trauma, hypersplenism, and splenic neoplasm, and have been extended further as the technique continued to improve [8]. In previously reported pediatric cases, most splenic hemangiomas were treated with partial or total splenectomy, and splenic embolization was infrequently performed [1, 2, 9]. In our case, there were no significant complications and only mild abdominal pain and intermittent fever occurred over the 7 days after splenic embolization. This was attributed to postembolization syndrome, which has been reported to be a common complication of splenic embolization and generally resolves without sequelae [7, 10].

Thrombocytosis has been observed 12 to 24 h after splenic embolization and the peak count is reached in 1 or 2 weeks [11]. The degree of thrombocytosis is positively correlated with the degree of spleen infarction and the decreased portal vein flow and increased platelet counts may result in portal vein thrombosis [7, 11]. In our case, the platelet counts reached a peak of 950,000/μL, and there were no thrombotic complications.

As most patients with splenic hemangioma are asymptomatic, an incidental abdominal mass is the most common clinical finding in children with splenic hemangioma. Some patients may have early satiety or abdominal pain due to a mass effect. In our case, although the patient had abdominal pain and diarrhea, it was associated with acute colitis rather than the large splenic hemangioma. There have been reports of heart failure, portal hypertension and gastrointestinal bleeding, thrombocytopenia, anemia, and consumptive coagulopathies such as Kasabach-Merritt syndrome associated with large splenic hemangiomas [1, 2, 4, 6]. The treatment of splenic hemangioma in children has been mostly partial or total splenectomy [1, 2], while Islam et al. reported that the use of oral prednisolone that had an effective antiangiogenic effect in a large splenic hemangioma in an infant [3]. Recently, propranolol has been used for primary therapy of infantile hemangioma; however, to our knowledge, there have been no reports of propranolol for splenic hemangiomas [12]. Further studies may be needed to determine the effectiveness propranolol for splenic hemangioma.

Although splenic embolization may have serious complications such as splenic abscess, splenic rupture, pancreatitis, and sepsis, it is a less invasive intervention and carries a reduced risk of bleeding compared with splenectomy. As our study was a case report, we cannot provide definite conclusion on the effectiveness of splenic embolization for large splenic hemangiomas. Therefore, further studies are needed to confirm the effectiveness of our approach.

## Abbreviation

CT: Computed tomography
